# Willingness to receive future COVID-19 vaccines following the COVID-19 epidemic in Shanghai, China

**DOI:** 10.1186/s12889-021-11174-0

**Published:** 2021-06-09

**Authors:** Yehong Zhou, Junjie Zhang, Wenwen Wu, Man Liang, Qiang-Song Wu

**Affiliations:** 1 The Changqiao Community Health Service Centre in Xuhui District, Shanghai, 200231 China; 2 Xuhui District Centre for Disease Control and Prevention, No. 50 Yong-Chuan road, Xuhui District, Shanghai, 200237 China; 3 The Huajing Community Health Service Centre in Xuhui District, Shanghai, 200231 China

**Keywords:** COVID-19, Epidemic, COVID-19 vaccine, Willingness, Knowledge

## Abstract

**Background:**

There are no pharmacological interventions currently available to prevent the transmission of SARS-CoV-2 or to treat COVID-19. The development of vaccines against COVID-19 is essential to contain the pandemic. we conducted a cross-sectional survey of Shanghai residents to understand residents’ willingness to be vaccinated with any future COVID-19 vaccines and take measures to further improve vaccination coverage.

**Methods:**

We conducted a cross-sectional survey using self-administered anonymous questionnaires from 1 July to 8 September 2020. The main outcome was willingness of participants, and any children or older individuals living with them, to receive future COVID-19 vaccines. Logistic regression analyses were used to explore potential factors associated with vaccination willingness.

**Results:**

A total of 1071 participants were asked about their willingness to receive future COVID-19 vaccines, for themselves and at least 747 children and 375 older individuals (≥60 years old) living with them. The highest proportion of expected willingness to vaccinate was among participants (88.6%), followed by children (85.3%) and older individuals (84.0%). The main reasons for reluctance to vaccinate among 119 participants were doubts regarding vaccine safety (60.0%) and efficacy (28.8%). Participants with a self-reported history of influenza vaccination were more likely to accept COVID-19 vaccines for themselves [adjusted odds ratio (*OR*) = 1.83; 95% confidence interval (*CI*): 1.19–2.82], their children (adjusted *OR* = 2.08; 95%*CI*: 1.30–3.33), and older individuals in their household (adjusted *OR* = 2.12; 95%*CI*: 1.14–3.99). Participants with older individuals in their families were less willing to vaccinate themselves (adjusted *OR* = 0.59; 95%*CI*: 0.40–0.87) and their children (adjusted *OR* = 0.58; 95%*CI*: 0.38–0.89).

**Conclusions:**

Participants were more reluctant to accept COVID-19 vaccines for older individuals living with them. The presence of older individuals in the home also affected willingness of participants and their children to be vaccinated.

## Background

Coronavirus disease 2019 (COVID-19), caused by severe acute respiratory syndrome coronavirus-2 (SARS-CoV-2), is responsible for a pandemic of more than 27.4 million confirmed COVID-19 cases and 894,983 deaths worldwide as of 9 September 2020 [1]. There are no pharmacological interventions currently available to prevent the transmission of SARS-CoV-2 or to treat COVID-19 [2]. Strict measures unprecedented in modern times have been implemented to contain the spread of the virus, including social distancing, stay-at-home orders, restrictions on travel and gatherings, and closures of schools and businesses [2]. These measures have had significant global impacts on social, cultural, and economic infrastructure [3–5].

Vaccines have been effective in controlling infectious disease epidemics [6, 7], and the development of vaccines against COVID-19 is essential to contain the pandemic and prevent new outbreaks. Fortunately, as of August 20, 2020, there are 30 candidate vaccines in clinical evaluation worldwide and an additional 139 candidate vaccines in preclinical evaluation [8]. Seven COVID-19 candidate vaccines are in clinical trials in China, three of which are in Phase 3 trials. The results of Phase 1 and 2 clinical trials showed that the candidate COVID-19 vaccines had good immunogenicity and safety [8–10]. As of March 29, 2021, five COVID-19 vaccines were approved for emergency use in china, including three inactivated vaccines, one adenovirus vector vaccine, and one recombinant subunit vaccine.

In China, the COVID-19 epidemic has been brought under control thanks to strong preventive and control measures [11]. Shanghai, one of China’s largest cities, experienced two stages of the COVID-19 epidemic: local epidemics (January to March, 2020) and overseas imports (March 2020 to present). As of 9 September 2020, a total of 342 local cases and 587 imported cases had been reported. However, multiple local outbreaks in northeastern China, Beijing, Xinjiang, Liaoning, and Guangdong, along with ongoing import of cases from overseas [12], put the vast majority of Shanghai residents with no immunity against SARS-CoV-2 at continued risk of infection. In response to the potential re-emergence of COVID-19 epidemics in Shanghai, it is necessary to understand residents’ willingness to be vaccinated with any future COVID-19 vaccines and take measures to further improve vaccination coverage. To our knowledge, there have been no surveys of Chinese residents’ willingness to vaccinate against COVID-19. Therefore, we conducted a cross-sectional survey of Shanghai residents to estimate the demand for future COVID-19 vaccines, providing a scientific basis for government decision-making.

## Methods

### Survey design

We conducted the survey from 1 July to 8 September 2020, about 3 months after the end of the local COVID-19 epidemic. During this period, imported cases from abroad continued to be reported. Adults and guardians of children (18–59 years old) who visited community health centers in Xuhui District, Shanghai, completed a questionnaire by scanning quick response (QR) codes with their phones during a 30-min waiting period for medical observation after completion of vaccination services. There are 13 community health centers in Xuhui District.

The survey was carried out using a self-administered, anonymous questionnaire consisting of four sections: (1) demographic information; (2) knowledge of COVID-19; (3) willingness to receive COVID-19 vaccines and reasons for acceptance or refusal of the vaccine; (4) responses on behalf of any children (0–18 years old) or older individuals (≥60 years old) living with them regarding willingness to receive COVID-19 vaccines. Survey responses were collected using Questionnaire Star software, a secure, web-based software used for survey research. Before the survey, participants were informed that only one adult per household was allowed to conduct the survey. Questionnaire need to be submitted before they leave the health center and could not be submitted repeatedly.

### Ethics and consent

This study was approved by the Ethics Review Board of Xuhui District Center for Disease Control and Prevention (No. XHCDC202001). The first page of the questionnaire included the consent form that explained the research project overview and participant’s confidentiality, making sure that their personal information would remain confidential and they hold the right to withdraw from the study whenever they wish to. Informed consent from participants was obtained prior to scanning the QR code to complete the questionnaire. Anonymity was guaranteed to participants. All methods were performed in accordance with the relevant guidelines and regulations.

### Statistical analysis

Descriptive analyses were used to describe demographic characteristics and knowledge of COVID-19. Differences among subgroups were assessed using Pearson’s Chi-square test. The expected willingness to vaccinate was calculated by weighting it with the registered residents of Shanghai in 2019 as the standard population. Univariate and multivariate logistic regression analyses were successively performed to explore potential factors (including sociodemographic information, COVID-19 knowledge, prospect of COVID-19 persistence, charges for future COVID-19 vaccines, and self-reported history of influenza vaccination) associated with the willingness of participants, children and older individuals to accept future COVID-19 vaccines. All statistical analyses were carried out using SPSS Version 18.0. Values of *p* < 0.05 or 95% confidence intervals (95% *CI*) excluding 0 were considered statistically significant.

## Results

### Participant characteristics

The average (± standard deviation) age of the 1071 participants who completed the survey was 34.0 ± 7.4 years. The majority of participants were female (76.5%) and had a Bachelor’s degree or higher (56.2%). Nearly half of participant households (48.3%) had more than four family members. A total of 747 participants had at least one child in their household, and 375 participants had at least one older individual living with them (Table 1).

**Table 1:** Demographic characteristics of participants (*N* = 1071)

Characteristic	Number	Percentage (%)
Gender		
Male	252	23.5
Female	819	76.5
Agee		
< 40	871	81.3
≥ 40	200	18.7
Healthcare-related occupations		
Yes	141	13.2
No	930	86.8
Level of education		
High school or lower	71	6.6
3-year college graduate	398	37.2
Bachelor’s degree or higher	602	56.2
Size of household		
1 to 3	554	51.7
≥ 4	517	48.3
At least one child in the household		
Yes	747	69.7
No	324	30.3
At least one older individual in the household		
Yes	375	35.0
No	696	65.0
Self-reported history of influenza vaccination		
Yes	403	37.6
No	668	62.4

### Participant’s knowledge of COVID-19 and outlook for COVID-19

The mean COVID-19 knowledge score of participants was 7.6 ± 1.4 out of 10 questions. Thus, participants had a relatively high level of COVID-19 knowledge; 80.1% of participants had scores ≥7. However, some participants scored low on questions regarding the source of COVID-19 infection (Table 2). A total of 914 participants (85.3%) identified close contact with individuals exposed to COVID-19 cases as the source of infection; 80 participants (7.5%) identified only confirmed COVID-19 cases as sources of infection; 16 participants (1.5%) identified only asymptomatic infected persons as sources of infection; and 38 individuals (3.5%) did not know the answer. In terms of COVID-19 outlook, 45.8% of participants believed it would persist, 27.3% believed it would be transient or short-term, and the remaining 26.9% were unable to assess future trends.

**Table 2:** Participants’ knowledge regarding COVID-19 (*N* = 1071)

Questions	Frequency of correct response.	Percentage (%)
Q1^†^. How many days of isolation are required after exposure to confirmed cases of COVID-19?	1017	95.0
Q2^‡^. Which measures can prevent COVID-19 infection?	1002	93.6
Q3^§^. Reusable masks can still prevent COVID-19 infection.	1001	93.5
Q4^§^. COVID-19 cases with chronic illnesses have a higher risk of a severe illness or death as an outcome.	979	91.4
Q5^†^. For COVID-19, who need to be isolated?	927	86.6
Q6^§^. People are generally susceptible.	904	84.4
Q7^†^. Where would you recommend a suspected cases of COVID-19 be treated?	866	80.9
Q8^†^. What are the main clinical symptoms of COVID-19?	768	71.7
Q9^†^. How is COVID-19 transmitted?	585	54.6
Q10^‡^. What are the sources of COVID-19 infection?	38	3.5

^†^Single-choice question; ^‡^Multiple-choice question; ^§^Decision-making question

### Willingness to receive COVID-19 vaccines

**Fig. 1:**
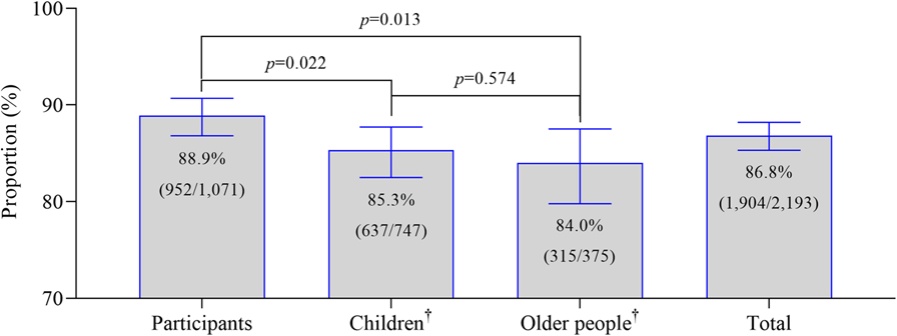
Proportions of participants, children, and older individuals willing to receive COVID-19 vaccines. ^†^Willingness of children and older individuals to receive COVID-19 vaccines was reported by participants living with them

In this study, 1071 participants decided for themselves and on behalf of at least 747 children and 375 older individuals whether to be vaccinated with future COVID-19 vaccines. In total, 1904 individuals (86.8%) were willing to be vaccinated; 88.9% of the 1071 participants were willing to be vaccinated themselves against COVID-19 if a vaccine became available, while 11.1% of respondents stated that they do not want to be vaccinated. The proportions of children and older individuals willing to receive COVID-19 vaccines were 85.3% (637/747) and 84.0% (315/375), respectively. Compared with their willingness to receive COVID-19 vaccines for themselves, participants were more reluctant to vaccinate children (88.9% vs. 85.3%; chi square = 5.2, *p* = 0.022) or older individuals (88.9% vs. 84.0%; chi square = 6.1, *p* = 0.013) in their households (Fig. 1). The excepted willingness to vaccinate was 86.7% among the population and 86.9% among adults after weighting with the population in Shanghai.

### Reasons for willingness and unwillingness to accept COVID-19 vaccines

**Fig. 2:**
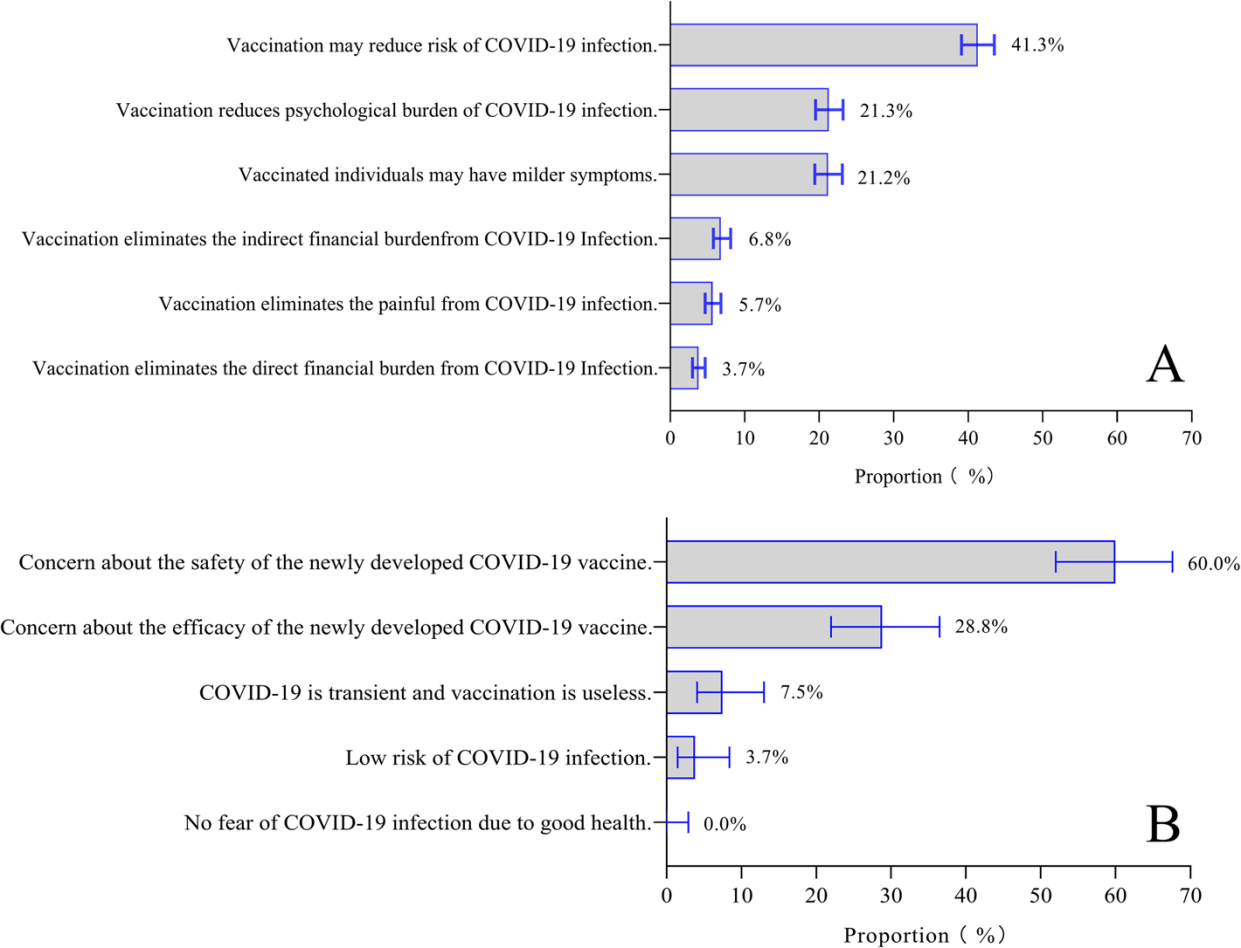
Reasons for participant willingness (**A**) and unwillingness (**B**) to receive future COVID-19 vaccines. Columns indicate the proportions of participants who reported the indicated reason for their willingness or unwillingness to vaccinate

The main reasons for 952 participants’ willingness to be vaccinated were reduced risk of COVID-19 infection (41.3%), reduced psychological burden of COVID-19 infection (21.3%) and less severe symptoms if COVID-19 infection occurs (21.2%). Furthermore, 16.2% of participants believed that vaccination could reduce pain and direct or indirect economic burdens arising from COVID-19 infection (Fig. 2A).

There were several reasons for the unwillingness of 119 participants to receive COVID-19 vaccines. Among these participants, 60.0% were concerned about the safety of a newly developed vaccine and 28.8% were concerned about its efficacy. Additionally, 7.5% doubted the necessity of vaccination and 3.7% believed that the risk of COVID-19 infection was low (Fig. 2B).

### Factors associated with willingness to receive COVID-19 vaccines

As shown in Table 3, self-reported history of influenza vaccination was positively associated with participants’ willingness to receive future COVID-19 vaccines for themselves (odds ratio (OR) = 1.74; 95%*CI*: 1.13–2.66), their children (*OR* = 1.77; 95%*CI*: 1.13–2.77), and older individuals in their households (*OR* = 2.14; 95%*CI*: 1.14–3.99). However, participants with older individuals in their households were less willing to vaccinate themselves (*O*R = 0.57; 95%*CI*: 0.39–0.83) and their children (*OR* = 0.56; 95%*CI*: 0.37–0.84). Participants with Bachelor’s degrees or higher were less willing to vaccinate themselves (*OR* = 0.29; 95%*CI*: 0.14–0.61) and their children (*OR* = 0.14; 95%*CI*: 0.05–0.4) than those with a high school education or lower. Participants with healthcare-related occupations were less likely to have their children vaccinated than other participants (*OR* = 0.51; 95%*CI*: 0.30–0.88).

**Table 3:** Factors associated with willingness of participants, children, and older individuals to receive COVID-19 vaccines in Shanghai, China

Variables	Willingness to vaccinate themselves (N = 1071)		Willingness to vaccinate children^†^ (*N* = 747)		Willingness to vaccinate older individuals^‡^ (*N* = 375)	
	% (n/N)	*OR* (95%*CI*)	% (n/N)	*OR* (95%*CI*)	% (n/N)	*OR* (95%*CI*)
Gender						
Male	86.5 (218/252)	Referent	83.6 (153/183)	Referent	85.9(85/99)	Referent
vFemale	89.6 (734/819)	1.35 (0.88–2.06)	85.8 (484/564)	1.19 (0.75–1.87)	83.3 (230/276)	0.82 (0.43–1.57)
Age						
< 40	89.0 (774/870)	Referent	84.9 (524/617)	Referent	82.4 (252/306)	Referent
≥ 40	88.5 (177/200)	0.95 (0.59–1.55)	86.9 (113/130)	1.18 (0.68–2.06)	91.3(63/69)	2.25 (0.93–5.47)
Healthcare-related occupations						
Yes	85.1 (120/141)	0.67 (0.40–1.12)	76.5(65/85)	0.51 (0.30–0.88)	82.4(42/51)	0.87 (0.40–1.90)
No	89.5 (832/930)	Referent	86.4 (572/662)	Referent	84.3 (273/324)	Referent
Level of education						
High school or lower	95.5 (168/176)	Referent	96.6 (112/116)	Referent	92.3(48/52)	Referent
3-year college graduate	91.1 (267/293)	0.49 (0.22–1.11)	89.8 (176/196)	0.31 (0.1–0.94)	81.1(73/90)	0.36 (0.11–1.13)
Bachelor’s degree or higher	85.9 (517/602)	0.29 (0.14–0.61)	80.2 (349/435)	0.14 (0.05–0.4)	83.3 (194/233)	0.41 (0.14–1.22)
Size of household						
1–3	89.2 (494/554)	Referent	86.4 (261/302)	Referent	82.9(92/111)	Referent
≥ 4	88.6 (458/517)	0.94 (0.64–1.38)	84.5 (376/445)	0.86 (0.56–1.3)	84.5 (223/264)	1.12 (0.62–2.04)
At least one child in the household						
Yes	88.9 (664/747)	1.00 (0.66–1.51)	/	/	85.8 (248/289)	1.72 (0.93–3.15)
No	88.9 (288/324)	Referent	/	/	77.9(67/86)	Referent
At least one elderly in the household						
Yes	85.1 (319/375)	0.57 (0.39–0.83)	80.6 (233/289)	0.56 (0.37–0.84)	/	/
No	90.9 (633/696)	Referent	88.2 (404/458)	Referent	/	/
Score of COVID-19 knowledge						
0–6	90.6 (193/213)	Referent	89.6 (121/135)	Referent	84.3(59/70)	Referent
7–10	88.5 (759/858)	0.79 (0.48–1.32)	84.3 (516/612)	0.62 (0.34–1.13)	83.9 (256/305)	0.97 (0.48–1.99)
Prospect of COVID-19 persistence						
Transient or short-term presence	90.8 (265/292)	Referent	89.6 (181/202)	Referent	88.9(88/99)	Referent
Persistent	87.1 (427/490)	0.69 (0.43–1.11)	81.7 (282/345)	0.52 (0.31–0.88)	81.2 (147/181)	0.54 (0.26–1.12)
Unable to judgement	90.0 (260/289)	0.91 (0.53–1.59)	87.0 (174/200)	0.78 (0.42–1.43)	84.2(80/95)	0.67 (0.29–1.54)
Charges for COVID-19 vaccines						
Yes	89.6 (309/345)	Referent	86.0 (203/236)	Referent	86.6 (110/127)	Referent
No	88.6 (643/726)	0.90 (0.60–1.37)	84.9 (434/511)	0.92 (0.59–1.42)	82.7 (205/248)	0.74 (0.40–1.35)
Self-reported history of influenza vaccination						
Unvaccinated	87.0 (581/668)	Referent	82.7 (383/463)	Referent	80.3 (184/229)	Referent
Vaccinated	92.1 (371/403)	1.74 (1.13–2.66)	89.4 (254/284)	1.77 (1.13–2.77)	89.7 (131/146)	2.14 (1.14–3.99)

Note: COVID-19: coronavirus disease 2019; CI: confidence interval; *OR*: odds ratio

^†^A total of 747 participants had at least one child in their household and made choices regarding COVID-19 vaccination on behalf of these children

^‡^A total of 375 participants had at least one older individual in their household and made choices regarding COVID-19 vaccination on behalf of these older individuals

Values indicated with a forward slash (/) were not suitable for statistical analysis

After adjustment for potential confounding variables, participants with a self-reported history of influenza vaccination remained more likely to choose the COVID-19 vaccine for themselves (adjusted O*R* = 1.83; 95%*CI*: 1.19–2.82), their children (adjusted *OR* = 2.08; 95%*CI*: 1.30–3.33), and older individuals in their household (adjusted *OR* = 2.12; 95%*CI*: 1.14–3.99). Participants with older individuals in their household remained less willing to vaccinate themselves (adjusted *OR* = 0.59; 95%*CI*: 0.40–0.87) and their children (adjusted *OR* = 0.58; 95%*CI*: 0.38–0.89). Participants with higher levels of education remained less willing to accept COVID-19 vaccines for themselves (adjusted *OR* = 0.29; 95%*CI*: 0.14–0.62) and their children (adjusted *OR* = 0.15; 95%*CI*: 0.06–0.43), and participants with healthcare-related occupations remained less likely to vaccinate their children (adjusted *OR* = 0.53; 95%*CI*: 0.30–0.94) (Table 4).

**Table 4:** Multivariable logistic regression analysis for the factors associated with willingness of participants, children, and older individuals to receive COVID-19 vaccines in Shanghai, China

Variables^†^	Willingness to vaccinate themselves (N = 1071)		Willingness to vaccinate children^‡^ (N = 747)		Willingness to vaccinate older individuals^§^ (N = 375)	
	Adjusted *OR*	95%*CI*	Adjusted *OR*	95%*CI*	Adjusted *OR*	95%*CI*
Level of education						
High school or lower	Referent		Referent		–	–
3-year college graduate	0.49	0.22–1.11	0.32	0.11–0.97	–	–
Bachelor’s degree or higher	0.29	0.14–0.62	0.15	0.06–0.43	–	–
Healthcare-related occupations						
Yes	–	–	0.53	0.30–0.94	–	–
No	–	–	Referent		–	–
At least one elderly in the household						
Yes	0.59	0.40–0.87	0.58	0.38–0.89	/	/
No	Referent		Referent		/	/
Self-reported history of influenza vaccine						
Unvaccinated	Referent		Referent		Referent	
Vaccinated	1.83	1.19–2.82	2.08	1.30–3.33	2.12	1.14–3.99

Note: COVID-19: coronavirus disease 2019; CI: confidence interval; *OR*: odds ratio

^†^In addition to these four factors in the Table 4, the multivariable logistic regression analysis also included participants’ gender, age, size of household, at least one child in the household, score of COVID-19 knowledge, prospect of COVID-19 persistence, charges for COVID-19 vaccines, and self-reported history of influenza vaccination

^‡^ A total of 747 participants had at least one child in their household and made choices regarding COVID-19 vaccination on behalf of these children

^§^A total of 375 participants had at least one older individual in their household and made choices regarding COVID-19 vaccination on behalf of these older individuals

Values that consist of a single hyphen (−) indicate the factor has no statistical significance in the model

Values indicated with a forward slash (/) were not suitable for statistical analysis

## Discussion

In the absence of effective control measures for COVID-19 [2], Shanghai’s high population density and general susceptibility of its residents makes it vulnerable to a renewed threat from COVID-19. In this study, we found that expected willingness to vaccinate against COVID-19 among Shanghai residents was high (86.7%). The proportion of participants willing to receive COVID-19 vaccines in our study was similar to that observed in two surveys conducted in Chile (90.6%) [13] and Australia (85.8%) [14], but higher than that observed in seven European countries (73.9%) [15] and in France (74%) [16]. However, willingness to accept COVID-19 vaccines in these surveys was substantially higher than willingness to vaccinate against H1N1 during the 2009 pandemic (8.7–67%) [17]. Most participants who were willing to be vaccinated believed that vaccination could reduce the risk or psychological burden of COVID-19 infection, as well as the likelihood of serious illness should infection occur. Because of the high risk of infection and high burden of COVID-19, there is strong willingness to vaccinate against COVID-19, although the severity of the COVID-19 epidemic varies among countries.

The herd immunity threshold, which describes the proportion of the population that needs to be immune to contain transmission, depends on the basic reproductive number (*R*_*0*_) of the disease [15]. Studies have reported values of *R*_*0*_ for SARS-CoV-2 ranging from 2.2 to 5.7 [18–20], and thus coverage would need to reach 54.5–82.5% for effective herd immunity [15]. Since vaccination programs are currently only for adults, proportion of expected willingness to vaccinate among adults was calculated. Although expected willingness to vaccinate among adults in this study was higher than the herd immunity threshold (82.5%), this is only an estimate of willingness to vaccinate and the actual coverage may be overestimated. Several studies showed that willingness to receive H1N1 vaccines was higher than the actual vaccination coverage during the 2009 H1N1 pandemic [21]. However, because H1N1 did not cause social consequences with the same magnitude as COVID-19 [22] and levels of awareness regarding the dangers of COVID-19 are likely much higher compared with H1N1, actual vaccination coverage may be increased. The results of a modeling study showed that coverage may be determined by the effectiveness of COVID-19 vaccines. As long as the effectiveness of the vaccine reaches 60–70%, 50–70% vaccination coverage can control the COVID-19 outbreak [23]. Because the safety and efficacy of a vaccine can greatly influence participants’ willingness to be vaccinated, the development of an efficient and safe COVID-19 vaccine is an urgent goal.

In this study, participants were more reluctant to accept vaccinations on behalf of older individuals in their households. However, the risk of serious disease and death following COVID-19 infection is higher in older individuals [24, 25]. Data from the United States indicated that 31% of cases, 45% of hospitalizations, 53% of intensive care unit admissions, and 80% of deaths associated with COVID-19 occurred among adults aged ≥65 years [25]. During the COVID-19 epidemic, family clusters were the main modes of human-human transmission accounting for 57.6% of all cases [26]. We found that participants with older individuals in their homes were less willing to vaccinate themselves and their children. In addition, participants with no self-reported history of influenza vaccination were less likely to accept COVID-19 vaccines for themselves, their children, and older individuals in their households. However, influenza vaccination coverage among Chinese residents was only 1.5–2.2% from 2004 to 2014, much lower than the proportion of participants’ self-reporting influenza vaccination history in our study [27]. Therefore, increasing the coverage of COVID-19 vaccines not only among older individuals, but also among their family members, will be required to prevent transmission within the family.

We also found that participants with healthcare-related occupations were more reluctant to have their children vaccinated against COVID-19. Furthermore, participants with higher levels of education were less likely to accept COVID-19 vaccines for their children. Children are less likely to have severe symptoms of COVID-19 infection [25]. However, children are at similar risk of infection as the general population, and mild or asymptomatic cases among children may also be sources of SARS-CoV-2 transmission [28]. Therefore, governments need to ensure that COVID-19 vaccination coverage in children is maintained at a high level once COVID-19 vaccine are available for children. Since vaccination programs are currently only for adults, children should become the focus of epidemic prevention and control with the implementation of vaccination among adults.

According to the Law of the People’s Republic of China on Vaccine Administration [29], a vaccine can be used on an emergency basis within a certain scope and time limit, with approval of governments, following a particularly significant public health event. An official from China’s health ministry said on television on 22 August 2020 [30] that China had initiated the emergency use of COVID-19 vaccines since 22 July. The purpose of emergency use is to ensure the stable operation of the city in the event of another COVID-19 epidemic by first vaccinating specific groups such as medical, epidemic prevention and border control personnel as well as personnel responsible for the basic operations of the city. Although there are several COVID-19 vaccines entering phase 3 clinical trials in China, approved vaccines may face initial undersupply challenges because of limitations in production capacity. To this end, Henn argues that in the absence of adequate supplies of future COVID-19 vaccines, the vaccine should be provided first to physicians and nurses as well as to police and other public security officers; second to organ transplant recipients; and finally to all others in order of date of birth from old to young, without exceptions [31]. This mirrors the vaccination strategy used in response to the 2009 H1N1 pandemic in China, in which priority groups (e.g., older individuals, students, civil servants, etc.) received the vaccine followed by other groups [32]. However, according to modeling results, the priority of targeted vaccination would depend on the effectiveness of future COVID-19 vaccines [23].

The study had several limitations. First, although the participants came from 13 community health centers in Xuhui district, the small size of participants may have represented a biased subset of Shanghai residents. Second, participants responded on their willingness to receive COVID-19 vaccines for their children or older individuals living in their households, which may not provide a true reflection of the willingness of children and older adults to be vaccinated. Third, our participants were highly educated and had received vaccines for themselves or their children, which may have overestimated our findings because they know more about vaccines. Fourth, the subjects were all from Shanghai, where the number of COVID-19 cases was small. Thus, our findings may not be fully generalizable to other regions. However, even among residents of Wuhan, the epicenter of the pandemic, only 2% had detectable IgM/IgG antibodies against SARS-CoV-2 [33]. Our study contributes novel and timely evidence to better understand the need for future COVID-19 vaccines following the COVID-19 epidemic. There are very limited data on this topic.

## Conclusion

Expected willingness to receive future COVID-19 vaccines among Shanghai residents was high (86.7%) following the COVID-19 epidemic. Willingness was higher than the highest predicted herd immunity threshold (82.5%). However, participants were more reluctant to receive COVID-19 vaccines for older individuals in their households. Furthermore, when there were older individuals in the home, it also affected willingness of the participants themselves and their children to accept vaccination. It is necessary for governments to increase the coverage of COVID-19 vaccines not only among older individuals but also for their family members; this is due to high rate of household transmission and the high risk for serious disease and death from COVID-19 among older individuals. When supplies of COVID-19 vaccines gradually become available in the future, the government must co-ordinate the allocation of priority vaccinations to ensure stable operation of the city in the event of another COVID-19 epidemic. At the same time, all countries must have access to effective vaccines to reduce the number of new infections, minimize burdens on healthcare systems, and reduce the social and economic impact of the COVID-19 pandemic.

## Data Availability

The datasets that support the findings of this study are available from the corresponding author on reasonable request.
